# Omega-3 Fatty Acids and Skeletal Muscle Health

**DOI:** 10.3390/md13116977

**Published:** 2015-11-19

**Authors:** Stewart Jeromson, Iain J. Gallagher, Stuart D. R. Galloway, D. Lee Hamilton

**Affiliations:** Health and Exercise Sciences Research Group, School of Sport, University of Stirling, Stirling, FK9 4LA Scotland, UK; E-Mails: stewart.jeromson@stir.ac.uk (S.J.); i.j.gallagher@stir.ac.uk (I.J.G.); s.d.r.galloway@stir.ac.uk (S.D.R.G.)

**Keywords:** fish oil, omega-3 fatty acids, skeletal muscle, hypertrophy and diabetes

## Abstract

Skeletal muscle is a plastic tissue capable of adapting and mal-adapting to physical activity and diet. The response of skeletal muscle to adaptive stimuli, such as exercise, can be modified by the prior nutritional status of the muscle. The influence of nutrition on skeletal muscle has the potential to substantially impact physical function and whole body metabolism. Animal and cell based models show that omega-3 fatty acids, in particular those of marine origin, can influence skeletal muscle metabolism. Furthermore, recent human studies demonstrate that omega-3 fatty acids of marine origin can influence the exercise and nutritional response of skeletal muscle. These studies show that the prior omega-3 status influences not only the metabolic response of muscle to nutrition, but also the functional response to a period of exercise training. Omega-3 fatty acids of marine origin therefore have the potential to alter the trajectory of a number of human diseases including the physical decline associated with aging. We explore the potential molecular mechanisms by which omega-3 fatty acids may act in skeletal muscle, considering the *n*-3/*n*-6 ratio, inflammation and lipidomic remodelling as possible mechanisms of action. Finally, we suggest some avenues for further research to clarify how omega-3 fatty acids may be exerting their biological action in skeletal muscle.

## 1. Introduction

The World Health Organisation estimates that in the last three decades global obesity has almost doubled to over 1.4 billion, meaning that as much as one third of westernised populations are classed as overweight/obese. Concurrent with the increase in obesity rates is an increase in obesity related disorders such as type 2 Diabetes (T2D), sarcopenic obesity and cardiovascular disease making obesity one of the major healthcare issues facing the world. Being overweight/obese increases the risk of developing diabetes by up to 30% [1]. The cost of primary diabetes care for the UK National Health Service (NHS) is currently at £9.8 billion/annum and is expected to continue rising (NHS). Skeletal muscle is a major site of glucose disposal, accounting for approximately 30% of postprandial glucose disposal [2]. Maintaining skeletal muscle metabolic health is therefore key to maintaining glycaemic control. Strategies that improve skeletal muscle metabolic function and insulin sensitivity could therefore have a major impact on the obesity induced development of insulin resistance and diabetes and reduce health care costs and improve quality of life. Skeletal muscle mass maintenance is not only crucial to the maintenance of metabolic function, but also through the control of locomotion it is also critical to the maintenance of physical function. Skeletal muscle (depending on adiposity levels) accounts for approximately 40% of total body mass and is highly adaptable to environmental changes such as diet and physical activity levels [3,4]. Loss of muscle mass with progressing age is an inevitable aspect of the aging process [5]. Reductions in skeletal muscle mass and metabolic function can have detrimental effects on overall health and is a major contributing factor to the onset of disease with age [6]. Loss of muscle mass and subsequent physical function not only places the individual at greater risk of chronic disease but leads to frailty and reduced quality of life [6]. From the age of approximately 50, skeletal muscle mass declines by 0.2%–0.5%/year and this loss is accelerated in a diseased state [7]. Moreover, as little as a 5% decrease in skeletal muscle mass has been associated with increased morbidity [8]. If the rate of sarcopenia (age related loss of muscle mass) can be reduced by 10% this would translate to a saving in US health care costs of $1.1 billion per year [9]. Therefore, it is of great clinical relevance to find effective therapies for improving muscle mass and metabolic function. Recent evidence suggests that manipulating the omega-3 polyunsaturated fatty acid (PUFA) content of skeletal muscle may improve muscle function and metabolism [10,11]. In this review, we will focus on the potential therapeutic role and molecular mechanism of action of omega-3 PUFAs, with an emphasis on marine derived omega-3 PUFAs, in the regulation of skeletal muscle metabolic and physical function. 

## 2. Dietary Fatty Acid Sources and the Influence of the *n*-3/*n*-6 Ratio on Metabolic Health

As previously mentioned, skeletal muscle is highly adaptable or mal-adaptable to changes in diet composition. In particular, diets high in saturated fat have been linked with the onset of both obesity and T2D [12,13]. However, diets high in polyunsaturated fatty acids such as the Mediterranean diet have been linked to beneficial outcomes, such as improved cardiovascular health [14,15]. Furthermore, the traditional diet of Inuit populations which is high in omega-3 PUFAs and low in omega-6 fatty acids is associated with a lowered risk of cardiovascular disease and improved insulin sensitivity despite being a diet very high in fat [16,17]. Therefore, the amount and type of fat in the diet can play an important role in regulating whole body metabolic health.

Fatty acid species are classified by their varying degrees of saturation into three main classes; saturated fatty acids (SFA), monounsaturated fatty acids (MUFA) and polyunsaturated fatty acids (PUFA). SFAs are a simple carbon chain containing no double bonds, MUFAs contain one double bond and PUFAs are classified as carbon chains containing two or more double bonds. The differences in the chemical structure of these different classes can lead to different physiological effects. For example, SFA has been linked with the development of metabolic dysfunction while conversely some MUFAs and PUFAs have positive effects on metabolic function [18,19].

At a cellular level, fatty acids are not only structurally important, as the main component of cellular membranes, but also have an important function in a number of metabolic processes such as regulating the activity of certain enzymes and by acting as signalling molecules [20]. Therefore, alterations in the composition of the muscle lipid pool may have profound effects on skeletal muscle metabolic and physical function. It is well established that skeletal muscle is sensitive to changes in dietary lipids, with a minimum 2-week alteration in dietary intake needed to significantly alter muscle lipid composition [21,22,23]. The change in cellular function brought about by changing lipid composition is possibly due to the fact that different species of fatty acids display a diverse array of structures ranging from simple saturated carbon chains to highly unsaturated carbon chains, these differences in structure are a large determinant of function. Before fatty acids can have an impact on cellular metabolism they must first be transported into the cell. However, fatty acid transport and metabolism must be tightly regulated as high intracellular levels of free fatty acids can be highly toxic due to lipid peroxidation, theoretically, highly unsaturated fatty acids will be most readily oxidized. As a result, there are a number of transporter proteins (CD36/FATP/FABP_pm_) and cytosolic proteins (FABP_c_) that regulate uptake and subcellular localisation of fatty acids, allowing for them to be stored or metabolised effectively [24]. Fatty acid uptake is analogous to glucose uptake, in which CD36 vesicles translocate from intracellular stores to the sarcolemma and is sensitive to insulin stimulation and contraction induced activation of AMPK [24]. These transporter proteins are also known to play a role in the regulation of mitochondrial fatty acid oxidation [25]. Once transported into the cell a number of intracellular fates are possible; mainly β-oxidation, storage in discrete lipid droplets or incorporation into cell membranes. In skeletal muscle the approximate ratio of storage to oxidation is 2:1 although this is variable depending on a number of factors such as energy expenditure and muscle fibre type [26]. Up to 90% of FAs transported into the cell are either stored or oxidized in soleus muscle [26]. Alternatively, fatty acids are incorporated into cellular membranes; the length of the carbon chain, number and position of double bonds of the fatty acids composing the membrane determine the physico-chemical properties such as fluidity [27]. Membranes which contain phospholipids lacking any double bonds pack tightly together reducing fluidity whereas the *cis*-kinks in the carbon chains caused by double bonds in unsaturated fatty acids pack less tightly and increase the deformability and fluidity of the membrane [28]. A change in membrane fluidity can alter the activity of membrane associated proteins, membrane receptors and vesicle budding and fusion [29,30].

The omega-3 fatty acids are a group of polyunsaturated fatty acids defined by a double bond at the third carbon from the methyl end of the carbon chain. Humans do not possess the necessary omega-3 desaturase to add a double bond at the 15th carbon of a long chain fatty acid and are, therefore, unable to endogenously synthesize alpha-linoleic acid (ALA 18:3*n*-3) and linoleic acid (LA 18:2*n*-6) making them essential fatty acids. Omega-6 PUFAs are also essential fatty acids and generally have metabolically distinct effects to omega-3 PUFAs. While the human body cannot synthesize omega-3 and omega-6 PUFAs, it does have the capacity to further metabolize these fatty acids through stages of elongation and desaturation. ALA can be metabolized to eicosapentaenoic acid (EPA 20:5*n*-3) and docosahexaenoic acid (DHA 22:6*n*-3) by ∆6 desaturase and ∆5 desaturase respectively, while LA is converted to arachidonic acid (AA 22:4*n*-6). However, the conversion of ALA to DHA is very inefficient with <10% conversion in females and <3% in males [31,32]. While ALA is the preferred substrate for ∆6 desaturase, an abundance of dietary linoleic acid has been shown to suppress conversion of ALA to DHA [33], which may be a confounding factor in these studies. There is recent evidence to suggest that supplementing with stearidonic acid (18:3*n*-3) may improve the efficiency of conversion to DHA, indicating ∆6 desaturase as a rate limiting step [34]. There is also a degree of individual variation in the lipidome following omega-3 supplementation in humans which may be a factor in the equivocal metabolic changes measured in many human supplementation trials [35].

It is thought that hominids’ diets during the Paleolithic era were high in seafood and low in seeds and vegetable oils, which led to an omega-3/omega-6 ratio of approximately 1:1 [36]. Given the likelihood that early human ancestors’ diets were already high in omega-3 intake it may not have conferred any evolutionary benefit to develop the ability to synthesize omega-3 PUFAs. During the agricultural revolution, with changes to food production in the Neolithic era this *n*-3/*n*-6 ratio began to diverge and now in the typical western diet is thought to be as much as 20:1, with omega-3 PUFA intake predominantly from ALA [37]. Although unlikely to be a primary driver, the divergence in the *n*-3/*n*-6 ratio has happened concurrently with the rise in CVD and states of chronic inflammation. Briefly, omega-6 PUFAs are associated with the production of pro-inflammatory mediators while omega-3 PUFAs produce less potent inflammatory mediators and inflammatory resolving proteins and so manipulating this ratio may bring about positive health outcomes.

The potential therapeutic benefit of a diet with high omega-3 content was first observed due to the lower incidence of CVD in Greenland Inuit populations [17]. Subsequent studies observed that a period of omega-3 supplementation reduced risk factors associated with CVD, such as the lowering of plasma triacylglycerides (TAGs) and an increase in high density lipoproteins at the expense of low density lipoproteins, as well as decreasing in platelet aggregation [35,38,39]. Yet, when end point measures such as cardiovascular disease are taken together in a meta-analysis the results of omega-3 supplementation are equivocal [40,41,42,43]. We hypothesize that while a given period of omega-3 supplementation leads to a significant increase in omega-3 content of various tissues it may not be sufficient to dramatically reduce the *n*-6/*n*-3 ratio.

The results of studies in animals in which the *n*-6/*n*-3 ratio is substantially reduced have been largely positive regarding insulin sensitivity and resolving inflammation. A particularly effective model for assessing the impact of *n*-6/*n*-3 ratios is the fat-1 transgenic mouse model which can endogenously synthesize omega-3 PUFAs from omega-6 PUFAs [44]. The ability to convert omega-6 fatty acids to omega-3 leads to an *n*-6/*n*-3 ratio of approximately 1. This model allows for the same diet to be used in all conditions and for the comparison of two significantly different *n*-6/*n*-3 ratios. It is, however, difficult to discern whether any effects are due to a reduction in the overall *n*-6/*n*-3 ratio or an increase in omega-3 PUFAs alone. Reductions in the *n*-6/*n*-3 ratio are associated with an improvement in whole body glucose tolerance, as well as preventing the age related decline in glucose tolerance [45,46]. Fat-1 mice were also protected from obesity related inflammatory activity and decrements in insulin sensitivity [47]. As well as improving glucose clearance, lowering the *n*-6/*n*-3 ratio also led to an increase in insulin secretion [48]. These studies demonstrate that a balance between omega-6 and omega-3 PUFAs within the lipid pool, may have a potential role in determining the metabolic effects of omega-3 PUFAs. Studies attempting to address this ratio in humans, however, would be difficult to adequately control. A human trial investigating a Mediterranean type diet which led to a reduction in the *n*-6/*n*-3 ratio observed that this reduction alongside other variables may confer some protection against metabolic dysfunction, providing some evidence that alterations in fatty acid content may have an effect on human health [15].

## 3. EPA *vs.* DHA

Omega-3 PUFAs consist of a heterogeneous mixture of fatty acids, of which eicosapentaenoic acid (EPA, 20:5) and docosapentaenoic acid (DHA, 22:6) are currently thought to be the most bioactive of the omega-3 species, however, docosapentaenoic acid (DPA, 22:5), an intermediary of EPA and DHA, may also have beneficial health effects [49]. Despite being very similar in structure and sharing some metabolic effects there is emerging evidence that different omega-3 PUFAs independently alter metabolic functions. The role of DPA in skeletal muscle metabolism remains unclear due to the only relatively recent availability in pure form. Yet, studies have observed that DPA has similar, and in some cases more potent, actions as EPA and DHA although to date the knowledge of DPA’s effect on skeletal muscle health is limited [50,51,52]. There is also evidence to suggest that EPA has a more potent effect on skeletal muscle protein metabolism compared with DHA [53]. The typical western diet, however, is deficient in omega-3 PUFAs and abundant in omega-6 fatty acids [54]. This *n*-6/*n*-3 ratio is linked to an increased state of chronic inflammation, which has been linked to diseases such as T2D and obesity [55]. There is now growing evidence to assert that, concurrent with CV and anti-inflammatory effects, omega-3 PUFAs play a beneficial role in skeletal muscle metabolism and function [10,11]. Although the mechanisms of action that underpin these changes remain to be fully characterised. We will discuss some of the studies demonstrating the mechanisms of omega-3 PUFAs and where applicable the potential differential effects of EPA and DHA.

## 4. Skeletal Muscle Anabolism

There is now growing evidence that omega-3 PUFAs also have intrinsic anabolic/anti-catabolic properties in skeletal muscle. Muscle protein balance is regulated by changes in the ratio of muscle protein synthesis (MPS): muscle protein breakdown (MPB). An increase in MPS or a decrease in MPB will lead to a positive balance and ultimately hypertrophy [56]. Muscle disuse due to illness or injury is associated with severe skeletal muscle loss [57]. However, omega-3 supplementation has been shown in some studies to blunt the loss of skeletal muscle mass [58,59,60]. It is well established that increasing amino acid availability stimulates a rise in MPS and omega-3 supplementation may potentiate this response to anabolic stimuli [61,62,63]. In a randomised controlled trial in healthy elderly individuals, it was observed that omega-3 PUFAs potentiated the muscle protein synthesis (MPS) response to simulated feeding (hyperinsulemia-hyperaminoacidemia clamp) following an 8-week supplementation period (1.86 g EPA, 1.5 g DHA/day), independent of increased glucose action. Importantly, omega-3 supplementation had no effect on basal rates of MPS [63]. A follow on study also observed the same trend in the effect of omega-3 PUFA on MPS in young and middle aged healthy individuals [62]. The increased MPS rates were independent of any anti-inflammatory activity as no changes in serum markers or plasma TAGs were observed following supplementation, this is likely explained by the fact that all volunteers were healthy individuals. Instead, this anabolic effect is thought to be partially mediated through an increased phosphorylation of mechanistic target of rapamycin (mTOR) and downstream signalling target P70-S6K1, a key regulatory pathway of protein synthesis and, by extension, muscle mass [64,65]. Upstream of mTORC1 no changes were detected in PKB in either study, suggesting a potential direct sensitisation of mTORC1 and P70-S6K1 to amino acid stimulation or alternatively an interaction of omega-3 PUFAs with other kinases involved in the activation of mTORC1. These studies suggest that omega-3 PUFAs improve muscle anabolism through an enhanced sensitivity to a nutritional stimulus likely independent of PKB activity.

A recent cell based study has suggested that EPA, and not DHA, is responsible for the anabolic effects of omega-3 PUFAs [53]. C_2_C_12_ myotubes treated with EPA showed elevated MPS 25% greater than the control with no effect of DHA despite P70-S6K1 phosphorylation being significantly elevated in both treatments [53]. These data would suggest that the effect of EPA on protein synthesis is likely independent of an enhanced anabolic signal through p70S6K1. Kamolrat *et al.* (2013) and others have shown that EPA may play a role in attenuating the rate of protein degradation [53,66,67,68]. The mechanism behind this effect appears to be through the inhibition of the NF-κB pathway [68]. EPA inhibits the degradation of the NF-κB complex by reducing phosphorylation of l κBα [69]. This action prevents translocation of NF-κB to the nucleus where it can induce expression of the muscle ring finger-1 gene (MURF-1) [66]. Knockdown of PPARγ significantly reduced the effect of EPA on NF-κB signalling. NF-κB is an important pathway linked with the loss of skeletal muscle mass as MURFF-1 facilitates ubiquitination of muscle proteins, effectively tagging them for degradation [70,71]. Animal models that lack MURF-1 expression are protected from atrophy [71,72]. Further support for a role of EPA in preserving muscle mass through inhibition of the atrophy pathways, comes from earlier work in a mouse cancer cachexia model. Treatment with EPA attenuated the loss of muscle mass through the suppression of the ubiquitin proteasome pathway [73]. Inhibition of this pathway could play an important role in the maintenance of skeletal muscle mass by offsetting periods of depressed MPS, a key contributing factor to atrophy. Contrary to other studies which observed no effect of DHA on protein breakdown, Wang *et al.* (2013) observed that DHA attenuated protein degradation through the same mechanisms as EPA but with greater efficiency [74].

There is *in vivo* evidence in humans that omega-3 PUFAs enhance the MPS response to feeding, and evidence from *in vitro* and rodent cancer models that omega-3 PUFA supplementation reduces muscle protein breakdown. Yet, it was unclear if long term omega-3 supplementation could improve muscle function. Rodacki *et al.* (2012) were the first to assess if omega-3 supplementation could improve muscle function in the elderly when combined with resistance training [11]. They supplemented 45 subjects for 90 days with 2 g/day omega-3 fatty acids in combination with a 90 day progressive resistance exercise program. They found that muscle strength and neuromuscular function was significantly improved when omega-3 supplementation was combined with resistance exercise, but omega-3 alone had no effect. A potential mechanism for omega-3 PUFAs improving contractile function may be an enhanced sensitivity of the muscle to acetylcholine, a neurotransmitter that stimulates muscle contraction. It has previously been observed that fish oil supplementation led to an increase in contractility in rats [75]. Strategies to improve or maintain neuromuscular function are of great interest as decrements in motor unit number and stability may precede functional changes in muscle [76]. Maintaining neuromuscular function may be an important factor in offsetting muscle loss, and there is some promising initial evidence that omega-3 PUFAs may be effective in maintaining both muscle mass and function in typically atrophic conditions. Indeed, a higher dose of omega-3 PUFAs than the dose used by Rodacki and colleagues (2012) demonstrated that supplementing elderly individuals with 4 g of omega-3 PUFAs daily for six months preserved muscle mass and function, not due to exercise induced effects, attenuating the normal declines associated with aging [10]. The mechanisms behind this preservation in muscle mass and function may be a combination of increases in postprandial MPS and improved neuromuscular function.

Despite several studies demonstrating the beneficial effect of EPA and DHA on muscle anabolism, a study in Sprague-Dawley rats suggested that omega-3 supplementation may blunt muscle recovery following a period of atrophy inducing immobilization [77]. The results showed that while fish oil blunted the loss of muscle mass during immobilization, muscle recovery was reduced at 3 days following remobilization, however, 13 days following remobilization muscle to body weight ratio and Myosin Heavy Chain had returned to similar levels. The early phase response inhibition was attributed to a reduction in PGF2α, a prostaglandin derived from arachidonic acid which has been shown to be important for muscle protein synthesis [78,79]. This may indicate that in healthy organisms without the presence of low grade inflammation that omega-3 PUFA supplementation may delay regeneration of muscle following damage.

Taken together, these studies suggest omega-3 PUFAs, particularly EPA, play a beneficial role in maintaining total protein balance (Table 1). It is thought that one of the main underlying causes of muscle wasting conditions, such as sarcopenia, is an impaired MPS response to anabolic stimuli [80]. Omega-3 PUFAs may, therefore, be of potential relevance to the clinical setting as a non-pharmacological method of reducing muscle loss. Further long term human trials are necessary to address whether long term omega-3 supplementation leads to muscle hypertrophy and consequent functional gains. Furthermore, the question remains whether omega-3 PUFAs not only improve the MPS response to nutrition but also increase MPS following an acute bout of resistance exercise. A recent study observed a rise in the total protein content in focal adhesion kinase, a mechanically sensitive protein, and mTOR which may indicate an enhanced capacity to respond to resistance exercise [23]. Experiments from tissue culture models indicate that EPA may be the significant contributor to changes in MPS rather than DHA, however, it has yet to be confirmed in humans if EPA underpins the changes in muscle mass and function.

**Table 1:** Summary of studies characterising the impact of omega-3 PUFAs on skeletal muscle metabolism.

Study	Model	Protocol	Key findings
Smith *et al.* (2011) [63]	Human	8 weeks, 1.86 g EPA, 1.5 g DHA daily	Augmented MPS and enahnced mTOR and p70S6K1 signalling in response to a hyperaminoacidemic-hyperinsulinemic clamp in young volunteer.
Smith *et al.* (2011) [62]	Human	8 weeks, 1.86 g EPA, 1.5 g DHA daily	Augmented MPS and enhanced mTOR and p70S6K1 signalling in response to hyperaminoacidemia-hyperinsulinemia in elderly volunteers.
Rodacki *et al.* (2012) [11]	Human	60 days pre training and 90 days during training, 0.4 g EPA, 0.3 g DHA	potententiated training increase in peak torque and rate of torque development (Knee extensor,flexor,plantar and dorsiflexor).
Smith *et al.* (2015) [10]	Human	6 months, 1.8 g EPA, 1.5 g DHA daily	Ameliorated age related declines in lean muscle mass. Increased hand grip strength and 1-RM muscle strength.
You *et al.* (2010) [59]	Rodent	14 days enriched with 5% cod liver oil followed by 14 days immobilisation	Reduced Myosin heavy chain loss during 14 days of hindlimb immobilsation.
Kamolrat *et al.* (2013) [81]	Rodent	8 weeks of choclate derived sweets, 49.6% EPA, 50.4% DHA	Increased phosphorylation of Pi3K and p70S6K1 during aminoacidemic-insulinemic clamp.
Gingras *et al.* (2007) [82]	Steers	5 weeks infusion 4% menhaden oil	Enhanced insulin action alongside an increase in amino acid disposal plus increased mTOR-p70S6K1 in response to hyperinsulinemic-euglycaemic-euaminoacidemic clamp.

## 5. Skeletal Muscle Metabolic Flexibility and Insulin Resistance

Skeletal muscle is highly adaptable to alterations in substrate availability and can switch between fat and glucose oxidation in response to changes in environmental conditions [83]. The ability to switch between different fuel sources, termed metabolic flexibility, is diminished by obesity and T2D at rest and during exercise [84,85]. Studies have observed that the ability to oxidize fat in particular is blunted by T2D [86,87]. Both EPA and DHA similarly improved the adaptability to changing substrate availabilities in cultured muscle cells [88]. However, not all studies examining the effect of omega-3 PUFAs on substrate selection have shown inconsistent results. In healthy individuals, three weeks of omega-3 supplementation increased fat oxidation by 35% at the expense of glucose utilization in response to a bolus of glucose [89]. While a follow up study indicated a tendency towards increased fat oxidation during exercise (60% VO2 max), however, differing modes and intensity of exercise would likely alter these results [90]. The shift towards fat utilization during exercise may be a characteristic of all PUFAs as omega-6 fatty acids suppress activation of pyruvate dehydrogenase (PDH) at the onset of exercise [91]. However, to the authors’ knowledge it has yet to be determined if omega-3 also suppresses PDH activity. Another group observed time dependent changes in substrate selection in T2D subjects with fish oil increasing glucose utilization after one week, however, after nine weeks fat oxidation was enhanced [92]. Yet, in diabetic but non obese rats EPA alone reduced total fat oxidation by 50%, instead fatty acids were directed towards storage [93]. Furthermore, a combination of EPA and DHA in rhabdomyosarcoma cells reduced the ratio between the oxygen consumption rate: extracellular acidification rate indicating a shift towards an increase on glycolytic reliance [94]. These data demonstrate a lack of consistency in an organism’s metabolic response to omega-3 PUFA supplementation.

There has been much debate on whether mitochondrial defects are a primary factor in the development of IR. Initial studies observed a reduction in mitochondrial content and size in insulin resistant and T2D individuals, accompanied by a reduction in expression of genes involved in mitochondrial biogenesis and oxidative phosphorylation [86,95]. Yet, it has also been reported that feeding rodents a short term high fat diet increases mitochondrial content and oxidative capacity despite the development of IR [96,97]. A number of studies have also reported normal mitochondrial function in insulin resistant populations [98,99]. Studies which have induced severe mitochondrial dysfunction actually observe an increase in insulin sensitivity. Despite these studies indicating that mitochondrial dysfunction is not a primary driver of IR, increases in mitochondrial content and oxidative capacity may help prevent the lipid accumulation associated with the onset of IR. There is some evidence that omega-3 PUFAs may stimulate mitochondrial biogenesis through the increased mRNA expression of transcription factors such as PCG1-alpha, TFAM and NRF1 [100,101]. This study observed an increase in mitochondrial abundance and oxidative capacity in response to a high fat diet without any added effect of fish oil [100]. Outwith, the context of a high fat diet 12 weeks of omega-3 PUFA supplementation (2 g EPA, 1 g DHA) did not alter mitochondrial abundance in humans, however, did increase ADP sensitivity [102]. It is unclear at present how the increased ADP sensitivity will alter mitochondrial function in the long term. In insulin resistant individuals omega-3 PUFAs did not alter mitochondrial abundance, maximal ADP respiration or peripheral insulin sensitivity [103]. In an aged mouse model replacing 3.4% of kcal with EPA but not DHA attenuated age related declines in mitochondrial function through the maintenance of mitochondrial protein quality, independent from changes in mitochondrial abundance [104]. There is also evidence that both LA and ALA in an obese animal model preserve insulin signalling and oxidative capacity through differential mechanisms [105]. Interestingly, the authors observed an increase in the abundance of electron transport chain sub-units in the subsarcolemmal mitochondrial pool independent of changes in mitochondrial content with ALA [105]. This study provides some evidence that some omega-6 PUFAs have similar protective effects to omega-3 PUFAs. The currently available data suggests that any insulin sensitising effects of EPA or DHA are unlikely to occur through increases in skeletal muscle mitochondrial abundance or oxidative capacity.

Skeletal muscle is a primary site of insulin stimulated glucose disposal and any dysfunction in this process can lead to the development of insulin resistance (IR), preceding T2D [2,106]. A reduced sensitivity of skeletal muscle to insulin stimulation is one of the primary defects leading to the development of T2D [107]. T2D is characterized in skeletal muscle by a reduction in glucose uptake, decreased glycogen synthesis, down regulation of fat oxidation and an expanded intramuscular (TAG) pool [108,109]. Lipid induced IR can be observed after only a few hours of increased plasma free fatty acids *in vitro* and in rodent skeletal muscle within three weeks of a high fat diet [110,111,112]. In particular, an overabundance of SFA (e.g., Palmitate) can induce IR through the accumulation of lipid derivatives including DAGs, ceramides and long chain acyl-coAs in the cytosol, which in turn can interfere with IRS1-PKB signalling [113,114,115,116,117,118]. However, our understanding of the role lipid metabolites have in a clinical context are still poorly understood. Increases in these lipid derivatives, particularly ceramides, can lead to the activation of PKC isoforms which lead to the phosphorylation of serine residues on insulin receptor substrate-1(IRS) and attenuating PKB activation [119,120]. In response to insulin, targets downstream of PKB initiate translocation of GLUT4, an insulin sensitive glucose transporter, to the membrane to facilitate transport. One of the prominent features of IR is the reduction of glucose uptake, caused by a reduction of GLUT4 at the cell surface despite no changes in total GLUT4 content [121,122].

Compared to SFA it appears that omega-3 PUFAs appear to at least have a neutral effect on skeletal muscle insulin sensitivity with some studies also showing a beneficial effect. While meta-analyses detect little or no impact of marine derived omega-3 PUFAs on insulin sensitivity or reduction in risk of developing T2D there are a number of human, animal and cell studies which support a potential role of omega-3 PUFAs in the treatment of IR/T2D through alterations in muscle metabolism, and omega-3 PUFAs may protect against some of the metabolic defects induced by a high fat diet [100,123,124,125]. A high omega-3 index (erythrocyte concentrations of EPA and DHA) is associated with higher insulin sensitivity and lower fasting insulin levels [126]. The results of current human clinical trials however are equivocal with some showing a beneficial effect [127,128] while some show no effect or adverse effects [103,129,130]. Yet, a recent study has observed that addition of omega-3 PUFAs to an intravenous lipid infusion attenuated the decline in insulin stimulated glucose action in muscle as well as an increased glucose disposal compared to an omega-6 infusion, these effects were independent of changes in acyl carnitine levels; a marker of mitochondrial overload [131]. During the infusion in this study intramyocellular lipids (IMCL) significantly increased by nearly 50% in the omega-3 group suggesting that omega-3 PUFAs promote the incorporation of fatty acids in complex lipids, instead of being directed towards β-oxidation [131]. Rodents fed a 10 week high fat diet with added fish oil attenuated the decline in glucose tolerance compared to a high fat diet and this effect was accompanied by a reduction in long chain acyl-coa species as well as in some ceramide species, although the abundance of these intracellular lipids were still elevated compared to the control diet [100]. These data may be explained by the findings that pre-treatment of C_2_C_12_ cells with EPA increased the incorporation of labelled oleate into TAGs and phospholipids, with no changes in β-oxidation observed [132]. Wensaas and colleagues (2008) also observed a marked increase in TAG synthesis following EPA treatment in isolated skeletal muscle myotubes and this effect was more pronounced in myotubes isolated from T2D individuals [133]. EPA also reduced the amount of C:18-2 CoA beyond that of control and again this effect was more pronounced in T2D myotubes. While increased levels of fatty acyl-CoA in general are associated with IR and in C18:2-CoA accumulation is a potential indicator of IR [115,117]. The ability of EPA to apparently increase incorporation of free fatty acids into TAGS may improve insulin sensitivity by preventing the accumulation of lipid intermediaries and the potential interference of these lipids on insulin signalling pathways. The potential for omega-3 PUFAs to increase complex lipid species in skeletal muscle is in contrast with data from other tissues where lipid droplet formation is suppressed [134]. Increasing intracellular TAGS has previously been shown to protect against lipotoxicity in states of lipid overload [135,136].

As well as increasing complex lipid formation, EPA may also improve both basal and insulin stimulated glucose uptake in a manner independent of PKB signalling [93,132]. The ability to clear blood glucose is diminished in IR/T2D individuals and sustained levels of high blood glucose can lead to negative health outcomes. Aas *et al.* (2005) observed that a 24 hour treatment of skeletal muscle cells with EPA increased basal glucose uptake 2.4 fold, however, the increase with insulin stimulation was similar to that of the control [132]. The changes in glucose uptake are likely independent of any changes in PKB signalling as Hessvik *et al.* (2010) and Aas *et al.* (2005) do not detect any differences in basal and insulin stimulated PKB phosphorylation [88,132]. One study observed that despite phosphatidylinositol-3-kinase (PI3K) activity being down regulated PKB phosphorylation remained intact in response to insulin [137]. Potentially indicating the omega-3 PUFAs may reduce the some signalling components within pathways. DHA is also known to play a role in glucose metabolism; preventing the deleterious effects of palmitate on PKB phosphorylation and glucose uptake [138]. Furthermore, increased transcription of genes involved in glucose regulation may also influence the metabolic effects of EPA and DHA. Coupled with an increase in glucose uptake observed by Aas *et al.* (2005) was an increase in mRNA content of GLUT1, which is involved in the regulation of basal glucose uptake [132,139]. Addition of fish oil to mice fed a high fat diet increased transcription of GLUT4 and IRS-1 [100]. Multiple studies have observed an increase in GLUT4 expression [93,94], however, whether this is reflected at the protein level is still unclear with one study showing no change in GLUT4 content with a low dose of omega-3 PUFAs [137]. These data suggest a potentially beneficial role for both EPA and DHA in glucose metabolism. Assessment of the effects of omega-3 PUFAs on cell and rodent models have returned largely positive results, however, this has yet to translate into consistent health benefits in human trials.

An alternative mechanism explaining the insulin sensitising effects of omega-3 PUFAs is through the reduction in inflammatory markers (see inflammation section). Briefly EPA and, to a lesser extent, DHA are natural ligands for Peroxisome proliferator-activated Receptor γ (PPARγ) and following activation of PPARγ NF-κB activity is suppressed reducing the release of pro-inflammatory cytokines [68]. Tumour necrosis factor-α (TNF-α) is also known to induce IR through the phosphorylation of IRS-1 on serine 307 similar to lipid intermediates [140] and EPA reduces TNF-α expression [93]. Furthermore, in macrophages and adipocytes the G-protein coupled receptor GPR120 is an omega-3 sensitive receptor that exhibits anti-inflammatory properties through the suppression of TNF-activation [141]. While this may be an important factor in clinical trials and rodent models, GPR120 expression is negligible in skeletal muscle and would therefore not explain the changes induced by omega-3 PUFAs in tissue culture models [141]. In rodents, knockout of AMPK subunit alpha 2 abrogates the improvement in hepatic insulin sensitivity by omega-3 PUFAs during a high fat diet despite studies observing little interaction between omega-3 PUFAs and AMPK [142]. It may be of interest to assess the importance of AMPK on insulin sensitizing effects of omega-3 PUFAs in skeletal muscle.

Taken together, these studies suggest that both EPA and DHA may have a protective effect against fatty acid induced insulin resistance, with some potential EPA or DHA independent effects. A summary of the results from human trials on glucose homeostasis can be seen in Table 2. These effects may be mediated by anti-inflammatory actions and the increased incorporation of potentially harmful fatty acid species into complex lipids, thereby reducing the interference with signalling pathways involved in glucose metabolism. Predominantly, fatty acid accumulation induces IR partially through a reduction in PKB related signalling and the data would suggest that EPA and DHA may protect against the blunting of PKB signalling rather than intrinsically increasing PKB activity. As such, several studies have shown improvements in glucose uptake with EPA, but not DHA, independent of changes in PKB phosphorylation. As well as potential improvements in peripheral insulin sensitivity, there is some evidence that omega-3 PUFAs may improve hepatic insulin sensitivity. Changes in hepatic insulin sensitivity are of great clinical relevance as the liver is a major contributor to postprandial glucose disposal. Lalia *et al.* (2015) and colleagues observed small reduction in endogenous glucose production while subset analysis of a meta-analysis indicated an increase in hepatic insulin sensitivity [103,124].

**Table 2:** Summary of studies characterising the impact of omega-3 PUFAs on glucose homeostasis.

Study	Model	Protocol	Key findings
Delarue *et al.* (1996) [89]	Human (healthy)	3 weeks, 1.1 g EPA, 0.7 g DHA daily	Reduction in insulinemia with an increase in non-oxidative glucose metabolism. Shift towards fat oxidation following a glucose load.
Delarue *et al.* (2006) [127]	Human (healthy)	3 weeks, 1.1 g EPA, 0.7 g DHA daily	Reduction in glucose fluxes during exercise (60% VO2 max). Tendency towards increase in fat oxidation during exercise.
Lalia *et al.* (2015) [103]	Human (insulin resistant)	6 months, 3.9 g EPA/DHA daily	No change in peripheral insulin sensitivity compared to control. Small reduction in hepatic gluconeogenesis.
Fasching *et al.* (1991) [129]	Human (impaired glucose tolerance)	2 weeks, 3.8 g EPA, 2.5 g DHA daily (30 ml fish oil)	No changes in fasting plasma glucose or insulin levels. No change in glucose or insulin during hyperinsulemic clamp.
Glauber *et al.* (1988) [130]	Human (T2D)	4 weeks, 18 g fish oil daily	Increase in fasting plasma glucose and in response to feeding. Increased hepatic glucose production. Reduction in insulin secretion.
Popp-snijders *et al.* (1987) [128]	Human (T2D)	8 weeks, 3 g EPA/DHA daily	Enhanced glucose clearance during steady state infusion of glucose and insulin.

## 6. Inflammation

Although an oversimplification, omega-6 fatty acids particularly arachidonic acid (AA, 20:4) have a more potent inflammatory effect compared to omega-3 PUFAs. While transient inflammation is an important process in muscle adaptation, failure to effectively resolve inflammation leading to a chronic state of inflammation is associated with IR/T2D and obesity [79,143]. There is growing evidence that omega-3 PUFAs have potent anti-inflammatory actions [144]. Synergistically, EPA and DHA play a role in the resolution of inflammation through the EPA and DHA derived inflammatory mediators such as prostaglandins, leukotrienes, lipoxins, resolvins and protectins. The anti-inflammatory effect of EPA and DHA are predominantly dependent on incorporation into phospholipids. EPA and DHA differentially alter the inflammatory response through the lipid specific production of lipid derived mediators. Eicosanoids are synthesised from 20 carbon fatty acid chains which have been released from phospholipids by phospholipase A_2_ meaning both AA and EPA are substrates for eicosanoid production. Incorporation of EPA and DHA into the substrate pool often occurs at the expense of AA reducing the potential for AA driven eicosanoid production. EPA, as a 20 carbon chain, competes with AA as a substrate for the cyclooxygenase (COX) and lypooxygenase (LOX) pathways and has also been seen to reduce the gene expression of COX-2 [145]. It is now also known that DPA also produces analogous inflammatory mediators to EPA and DHA, and elongation of EPA to DPA may be an important event in the inhibition of the COX pathway [52,146]. Dietary supplementations of omega-3 PUFAs in humans have observed decreased production off AA eicosanoids such as Prostaglandin E_2_ [147,148]. EPA derived eicosanoids also display a reduced affinity for eicosanoid receptors rendering them up to 50%–80% less biologically active than AA derived eicosanoids [149]. Both EPA and DHA also serve as substrates for resolvins, protectins and maresins which display inflammation resolving properties in both cell culture and animal models of inflammation [150,151]. However, a recent study has called into question the production of inflammatory resolving molecules and their role in mediating inflammatory challenges in healthy humans [152]. A short term supplementation period of 17.6 g EPA and DHA/day did not result in the detection of these inflammatory resolving mediators in plasma or urine [152]. Further study will be necessary to understand whether endogenous production of resolving molecules has a prominent role in the inflammatory process.

As well as altering eicosanoid production, omega-3 PUFAs can also reduce activation of the NF-κB pathway, reducing inflammatory cytokine production contrasting the omega-6 fatty acid AA which is a known stimulator of NF-κB activity [153]. Omega-3 PUFAs prevent the degradation and subsequent translocation of the NF-κB complex to the nucleus where it induces transcription of inflammatory cytokines. The reduction in NF-κB pathway activation is thought to be caused by an up-regulation in PPARγ activity, which is known to interfere with NF-κB activation [154]. As a result studies have observed reductions in circulating TNF-α concentrations (which may also provide negative feedback on NF-κB activity), as well as expression of inflammatory cytokines and cell surface adhesion molecules [155,156,157]. Although inflammation may not have a primary role in the development of IR in skeletal muscle, it may accentuate the metabolic dysfunction caused by the onset of IR/T2D [110]. For instance, conditioned media from palmitate treated macrophages induces insulin resistance in skeletal muscle cells [158]. Furthermore, a number of animal studies where inflammatory pathways are genetically down regulated demonstrate that preventing obesity induced inflammation can prevent the development of insulin resistance [159,160]. Controlling inflammation may, therefore, be an important factor in the long term management of skeletal muscle IR. *In vivo* studies assessing the immunomodulatory effect of omega-3 PUFAs in humans are limited. However, a diet high in EPA and DHA is inversely associated with reactive-c protein concentrations [161]. Omega-3 PUFAs have also been shown to increase lymphocyte proliferation as well as altering neutrophil and natural killer cell function [162,163,164,165]. The results of *in vivo* studies are mixed with some showing a beneficial effect in adipose tissue of severely obese individuals while no effect was seen in moderately overweight individuals [166,167]. Exercise can also have an impact on immune function and six weeks omega-3 supplementation augments natural killer activity post exercise [168]. The research on the necessary dose to elicit an anti-inflammatory effect is still limited. One study found that the ability of EPA to reduce Prostaglandin E2 occurred between an intake of 1.35 and 2.75 g/day, suggesting a threshold for the anti-inflammatory effects [148]. The dose of EPA is typically lower in many studies which may be a possible factor in the equivocal changes in inflammatory markers seen in supplementation studies. Given that skeletal muscle accounts for up to 40% of total body mass, a significant change in lipid composition and subsequent eicosanoid production may have a large influence on systemic inflammatory status.

## 7. Remodelling the Lipidome

Cellular membranes are a complex combination of lipids, cholesterol and proteins which allow for the compartmentalization of chemical processes and are sensitive to dietary fatty acids. Omega-3 PUFA incorporation leads to a remodelling of cellular membranes and is not limited to the plasma membrane as dietary omega-3 PUFAs have been shown to alter subcellular membrane composition such as in mitochondria [102]. Several studies have observed a significant enrichment in the muscle total lipid pool and phospholipids by omega-3 PUFAs with an approximate two fold increase during a supplementation period of between four and 12 weeks [21,23,62,63]. Given that omega-3 PUFAs interact with multiple cell signalling pathways changes in the membrane composition may be a potential convergence point for the effects of omega-3 PUFAs on metabolic outputs. The phospholipid pool also serves as the main substrate pool for production of eicosanoids. Enriching the phospholipid fraction specifically may, therefore, be necessary to elicit the beneficial effects on omega-3 PUFAs. To date, few studies have fully characterised to what extent, or even which, phospholipid fractions (*i.e.*, phosphatidylcholines, phosphatidylerines, phosphatidylethanolamines and phosphatidylinositol) are enriched or displaced by omega-3 PUFAs in skeletal muscle and how this relates to skeletal muscle metabolism. A study by Rossmeisl and colleagues (2012), observed favourable metabolic changes when omega-3 PUFAs were available as phospholipids compared with TAGs, possibly due to different bio-availabilities [169]. Alterations in the membrane composition within cells are known to alter the physico-chemical properties. An increase in unsaturation of phospholipids will increase membrane fluidity whereas greater saturation of phospholipids will induce a more rigid structure. As such, insulin resistance is associated with decreasing concentration of PUFAs in serum and muscle phospholipids [170]. Theoretically, enhanced fluidity of the membrane may facilitate increased GLUT4 vesicle fusion with the plasma membrane, however, it would not explain why EPA and DHA would differentially regulate glucose uptake. Membrane composition changes may alter the micro-environment of proteins localized within the membrane causing a modification in the function of the protein [29]. There is also evidence to suggest that DHA, and to a lesser extent EPA, play a role in the formation and composition of lipid rafts [171]. Lipid rafts are insoluble fractions of the membrane composed of sphingolipids, cholesterol and proteins and are formed in order to allow the compartmentalisation of the membrane for the coordination of function by providing scaffolding to stabilise protein-protein and lipid-lipid interactions involved in signal transduction and trafficking [172]. Studies have shown in a number of cell lines that DHA and EPA are incorporated into insoluble membrane fractions and lead to the ‘declustering’ of lipid rafts due to the poor affinity between omega-3 PUFAs and cholesterol, leading to the expulsion of cholesterol from the lipid rafts [171,173]. However, EPA and DHA may have divergent effects on the structure of lipid rafts with EPA incorporated into non-raft regions and displacing cholesterol to raft regions making them more ordered, while DHA may have the opposite effect [174]. These structural changes may affect protein localisation within raft and non-raft regions, however, it is yet to be fully understood how this directly impacts on cell function. Given the important nature of signalling responses at the membrane, modification by omega-3 PUFAs could lead to a significant alteration in cellular function giving rise to changes in gene expression, cell signalling and release of bioactive metabolites. The current literature, however, focuses on immune cell lines and it remains to be studied in human skeletal muscle cells. It is also unknown if omega-3 incorporation may differ between fibre types and if this is related to fatty acid turnover rates.

## 8. Considerations for Future Research

Despite rodent and tissue culture studies displaying that EPA and/or DHA are metabolically active in skeletal muscle, questions still remain about the effectiveness in humans, particularly regarding inflammation and insulin sensitivity. There are a number of variables that have likely influenced the wide range of different results. To date, no consensus has been reached on what constitutes an effective dose of omega-3 PUFA, a question likely confounded by individual variation and no apparent dose-response relationship. Previous studies have observed individual rate uptakes of omega-3 PUFAs into tissues [35], so the same supplementation period and dose may lead to different tissue levels of omega-3 PUFAs between subjects, potentially masking any effect of elevating omega-3 PUFA tissue levels. Both the supplementation period and length of follow up measures need to be taken into consideration as these factors will influence outcome measurements. For example, Mostad and colleagues (2006) observed that the short term response to intake of omega-3 PUFAs was a decrease in fat oxidation but the opposite effect was observed after nine weeks [92]. We hypothesize that manipulation of the *n*-3/*n*-6 ratio may be an important factor in maximising the effects of omega-3 PUFAs, however, whether studies are looking to examine this effect or the isolated effect of omega-3 PUFAs, make placebo choice key. Studies using a high omega-6 corn oil as a control may also have metabolic effects and may not represent a true placebo.

## 9. Conclusions

**Figure 1:**
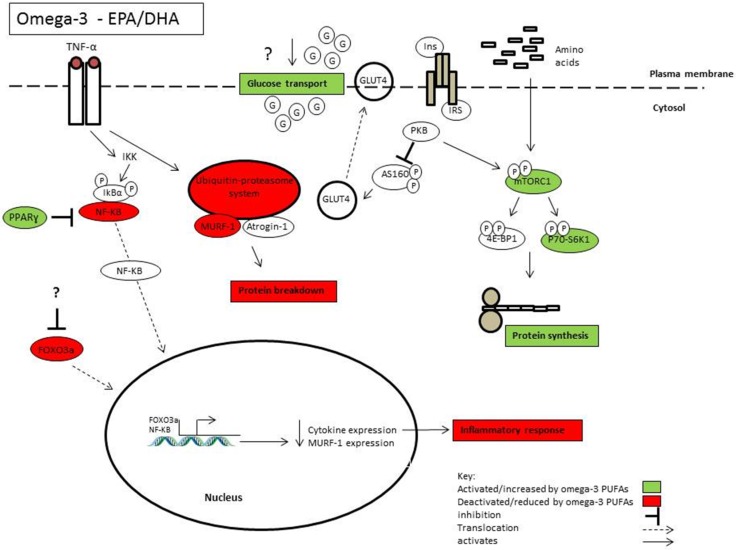
Highlights some of the mechanisms of action by which omega-3 PUFAs EPA and DHA may influence skeletal muscle health and function.

The results from omega-3 supplementation trials have often been mixed, yielding null responses when combined in meta-analyses. We hypothesize that one of the primary variables that may limit the effect of omega-3 fatty acids is a high *n*-6/*n*-3 ratio. Evidently the relationship between omega-3 and omega-6 PUFAs is not simply antagonistic and different effects of fatty acids within each subclass adds to the complexity. Further studies should aim disseminate the interaction between omega-3 and omega-6 PUFAs and how this may impact metabolism. The currently available evidence suggests that omega-3 PUFAs EPA and DHA may be effective in preventing the deleterious effects of atrophic conditions or low grade inflammation (Figure 1). The research has focused predominantly on EPA and DHA, yet it is now emerging that DPA has also been shown to have similar, and in some cases, more potent effects. There is emerging evidence that different omega-3 PUFAs has divergent metabolic functions, and further research is required to understand the different mechanisms underpinning these effects. The recent advances in the “omic” techniques and mass spectrometry technology will allow for a comprehensive and sensitive approach to analysing the metabolic changes induced through omega-3 uptake. Currently, omega-3 PUFAs are mainly derived from marine sources. Given the increasing environmental pressures on fish populations it brings into question the sustainability of fish as a suitable resource of omega-3. It is estimated by 2050 that the human population will reach 9.1 billion and global warming will reduce the omega-3 content in algae, reducing the total omega-3 content in fish [175]. This highlights the need to understand the mechanisms of omega-3 PUFA action which may lead to the development of an omega-3 mimetic and provide a sustainable long term source.
